# C1GalT1 expression reciprocally controls tumour cell-cell and tumour-macrophage interactions mediated by galectin-3 and MGL with double impact on cancer development and progression

**DOI:** 10.1038/s41419-023-06082-7

**Published:** 2023-08-23

**Authors:** Yangu Wan, Kareena Adair, Anne Herrmann, Xindi Shan, Lijun Xia, Carrie A. Duckworth, Lu-Gang Yu

**Affiliations:** 1 Department of Biochemistry, Cell and Systems Biology, Institute of Systems, Molecular and Integrative Biology, University of Liverpool, Liverpool, UK; 2 Centre for Proteome Research, University of Liverpool, Liverpool, UK; 3 Department of Molecular and Clinical Cancer Medicine, Institute of Systems, Molecular and Integrative Biology, University of Liverpool, Liverpool, UK; 4 Oklahoma Medical Research Foundation, Oklahoma City, OK, USA; 5 Department of Molecular Physiology and Cell Signalling, Institute of Systems, Molecular and Integrative Biology, University of Liverpool, Liverpool, UK

**Keywords:** Cancer microenvironment, Glycobiology

## Abstract

Although most cell membrane proteins are modified by glycosylation, our understanding of the role and actions of protein glycosylation is still very limited. β1,3galactosyltransferase (C1GalT1) is a key glycosyltransferase that controls the biosynthesis of the Core 1 structure of O-linked mucin type glycans and is overexpressed by many common types of epithelial cancers. This study reports that suppression of C1GalT1 expression in human colon cancer cells caused substantial changes of protein glycosylation of cell membrane proteins, many of which were ligands of the galactoside-binding galectin-3 and the macrophage galactose-type lectin (MGL). This led to significant reduction of cancer cell proliferation, adhesion, migration and the ability of tumour cells to form colonies. Crucially, C1GalT1 suppression significantly reduced galectin-3-mediated tumour cell-cell interaction and galectin-3-promoted tumour cell activities. In the meantime, C1GalT1 suppression substantially increased MGL-mediated macrophage-tumour cell interaction and macrophage-tumour cell phagocytosis and cytokine secretion. C1GalT1-expressing cancer cells implanted in chick embryos resulted in the formation of significantly bigger tumours than C1GalT1-suppressed cells and the presence of galectin-3 increased tumour growth of C1GalT1-expressing but not C1GalT1-suppressed cells. More MGL-expressing macrophages and dendritic cells were seen to be attracted to the tumour microenvironment in ME *C1galt1*^-/-^/Erb mice than in *C1galt1*^f/f^ /Erb mice. These results indicate that expression of C1GalT1 by tumour cells reciprocally controls tumour cell-cell and tumour-macrophage interactions mediated by galectin-3 and MGL with double impact on cancer development and progression. C1GalT1 overexpression in epithelial cancers therefore may represent a fundamental mechanism in cancer promotion and in reduction of immune response/surveillance in cancer progression.

## Introduction

Protein glycosylation is one of the commonest post-translational processes. Glycosylation of proteins not only allows the proteins to be adequately folded for designated functions, the carbohydrate residues that are added to the proteins are often also directly involved in cell recognition by interaction with other molecules such as carbohydrate-binding proteins (lectins) in the microenvironment [1, 2].

Most, if not all, cell membrane proteins are glycoproteins that are modified with various length and number of carbohydrate residues [2]. One of the most common types of protein glycosylation is the mucin type O-linked glycosylation (O-glycosylation). This type of protein glycosylation occurs through sequential actions of an array of glycosyltransferases, starting from the action of one of 20 polypeptide *N-*acetyl-galactosaminyltransfersases (GALNTs). GALNTs catalyses the addition of a GalNAc residue to serine or threonine (Ser/Thr) residues on the protein backbone for the formation of GalNAcα-Thr/Ser (Tn antigen) [3]. The GalNAc residue is then competed by the actions of three other types of glycosyltransferases for further carbohydrate chain elongation or chain termination. It can be modified by β1,3galactosyltransferase (C1GalT1, T-synthase) for the addition of a galactose residue to form the Core 1 structure Galβ1, 3GalNAcα-Ser/Thr (TF antigen), or by a GlcNAc-transferase (β3GnT) for the addition of a GlcNAc residue to form the Core 3 structure of GlcNAc β1,3GalNAcα-. Both Core 1 and 3 structures are modified further by other glycosyltransferases to form long carbohydrate structures [4, 5]. The GalNAc residue can also be modified by a sialyltransferase (ST6GalNAc-T) for the formation of sialic acid-GalNAcα-Thr/Ser (Sialyl-Tn antigen) for chain termination [4]. While there are normally multiple glycosyltransferases for catalysation of the addition of a monosaccharide residue in the glyco-biosynthesis machinery, C1GalT1 is known to be the sole glycosyltransferase to catalyse the addition of galactose to the GalNAc for the formation of Core-1-associated carbohydrate structures [6].

Changes of protein glycosylation such as O-glycosylation occurs commonly in cancer and pre-cancerous conditions [7, 8]. Many of these O-glycosylation changes lead to the appearance of short oncofetal carbohydrate structures such as Tn, sialyl-Tn and TF antigens which are normally concealed by other carbohydrate structures under physiological conditions [2]. These short chain oncofetal carbohydrate structures are seen in over 90% epithelial cancers [9] and their appearances are often correlated with poor prognosis and poor survival of patients [4, 10]. Many of these short-chain carbohydrate structures are recognised by endogenous lectins such as the galactoside-binding galectins [11], GalNAc-binding MGL [12] and sialic acid-binding siglecs [13].

More and more studies have demonstrated close association of protein glycosylation changes in cancer progression and metastasis [14–16]. For example, tumours from 90% breast cancer and 75–90% pancreatic ductal adenocarcinoma show substantial changes of protein O-glycosylation and these changes are correlated with advanced metastasis and poor prognosis [8, 17, 18]. Although the mechanisms that underpin the changes of protein glycosylation in cancer are complex and often involve multiple factors such as changes of Golgi pH [19] and alteration of endoplasmic reticulum-localised molecular chaperons [20], changes of the expression of glycosyltransferase are believed to be one of the key determinants in cancer [4]. As the sole glycosyltransferase that catalyses the formation of Core 1-associated glycans during O-glycosylation, C1GalT1 is commonly overexpressed in various cancers such as colon [21], breast [22], gastric [23], head and neck cancers [24]. Over expression of C1GalT1 correlates with high malignancy, poor prognosis and poor survival of cancer patients [6, 21–23]. We hypothesized that overexpression of C1GalT1 by tumour cells would change the appearance of short-chain carbohydrate structures such as the unsubstituted TF or Tn and this may alter tumour cell-cell and tumour-stromal cell interactions mediated by the galactoside-binding galectin-3 and GalNAc-binding macrophage lectin (MGL) in the tumour microenvironment (TME), with critical consequence in cancer progression.

Galectin-3 and MGL are both known active regulators in cancer progression [25]. Galectin-3 is commonly overexpressed in most types of cancer [25] and promotes multiple steps in cancer progression and metastasis such as cancer cell adhesion, invasion [26] and angiogenesis [27] by interaction with galactose-terminated cell surface or extracellular matrix glycans [11]. MGL is a Ca^2+^ dependent GalNAc binding lectin [12] and is expressed predominately by macrophages and dendritic cells (DCs) [28, 29]. MGL can bind to CD45 on effector T cells and reduce effector T cell proliferation [30] and decrease T cell-mediated anti-tumour actions.

A series of analyses were conducted in this study and showed that alteration of C1GalT1 expression in human colon cancer cells reciprocally changes tumour cell-cell and tumour-macrophage interactions mediated by galectin-3 and MGL with significant impact on tumour growth and progression.

## Materials and methods

Antibodies against C1GalT1 (F-31, sc-100745) and p-EGFR (F-3, sc-377547) were purchased from Santa Cruz Biotechnology (Texas, USA). Biotinylated-peanut agglutinin (PNA, B-1075), -vicia villosa lectin (VVA, B-1235-2), -maackia amurensis lectin-II (MAL-II, B-1265-1), -sambucus nigra lectin (SNA, B-1305-2) and -griffonia simplicifolia lectin-II (GSL-II, B-1215-2) were purchased from Vector Lab (California, USA). Anti-EGFR (D38B1, 4267 S) monoclonal antibody was purchased from Cell Signalling Technology (Ledien, Netherlands). CellTiter 96 AQ_ueous_Non Radioactive Cell Proliferation Assay (MTT, colorimetric) and CellTiter-Glo® Luminescent Cell Viability Assay (ATP) kits were from Promega (Southampton, UK). Recombinant human CLEC10A/CD301, monoclonal antibody against human/mouse CLEC10A/CD301 (AF4888) and human galectin-3 biotinylated antibody (BAF1154) were from R & D Systems (Abingdon, UK). Three- well Culture-Inserts were from Ibidi GmbH (Munich, German). Matrigel® Basement Membrane Matrix (Phenol Red Free) was purchased from CORNING, (New York, USA). Phorbol 12-myristate-13-acetate (PMA), lipopolysaccharides (LPS) from *Escherichia coli* O111:B4 and anti-Actin antibody (A2066) were from Sigma-Aldrich (Gillingham, UK). Recombinant human IFN-γ, IL-4 and EGF were from PeproTech (London, UK). Vybrant^TM^ DiO Cell-labelling solution and Vybrant^TM^ DiI Cell-labelling solution, monoclonal antibodies to PE-CD68 (eBioY1/82 A, 12-0689-42), PE-CD86 (12-0869-41), PE-EGR2 (12-6691-82) F4/80 (14-4801-82) and CD11c (14-0114-82) were from Thermo Fisher Scientific (Glasgow, UK). C1GalT1 polyclonal antibody (BS-9685R) was purchased from Bioss (Edinburgh, UK). EDTA-free Protease inhibitor Cocktail was from Roche (Basel, Switzerland). Fertilised Shaver Brown hen eggs were obtained from Henry Stewart & Co. Ltd, UK (medeggs.com).

### Cell lines

HCT116F3shC1GT and HCT116F3shCon were human colon cancer HCT116F3 cells [31] that were transfected with C1GalT1 shRNA and control shRNA respectively [7]. HCT116F3 transfected cells were cultured in McCoy’s 5 A glutaMAX^TM^ medium containing 10% foetal calf serum (FCS) and 1% penicillin-streptomycin. SW620shC1GT and SW620shCon cells were human colon cancer SW620 cells that were transfected with C1GalT1 shRNA or control shRNA, respectively [32]. ShRNA plasmid DNA for Core 1 Gal-transferase (SHCLND-NM_020156-C1GALT, TRCN0000289384), control shRNA (SHC002v) were from Sigma Aldrich. SW620 transfected cells were cultured in DMEM glutaMAX^TM^ medium containing 10% FCS and 1% penicillin-streptomycin. THP-1 cells (kindly provided by Dr. Jack Zhang, University of Liverpool) were cultured in RPMI1649 medium containing 10% FCS, 1% penicillin-streptomycin.

The cell lines were last authenticated in 2020 by DNA profiling at the Cell Line Authentication Facility at University of Liverpool.

### Lectin/Immuno-blotting

Seventy percent confluent cells were lysed by SDS sample buffer (0.5 M Tris-HCl at pH 6.8, glycerol, 2-mercaptoethonal, 10% SDS). Cell lysates were applied to SDS-PAGE and transferred to nitrocellulose membrane. The membrane was incubated with blocking buffer [1% Bovine Serum Albumin (BSA) in PBS] for 50 min before incubation with antibody against C1GalT1 (0.1 µg/ml), biotinylated-SNA (1.5 µg/ml), -PNA, -VVA or -GSL-II (0.6 µg/ml), or with antibody against p-EGFR (0.1 µg/ml) in blocking buffer overnight at 4 °C. The blots were washed three times with 0.1% Tween-20 in PBS before incubation with peroxidase-conjugated secondary antibody (1:8000) or peroxidase-avidin (1:8000) for 1 h. After six washes with PBS-0.1% Tween-20, the blots were developed with SuperSignal^TM^ West Dura Extended Duration Substrate (Thermo scientific^TM^) and visualised with Molecular Imager Gel Doc XR System (Bio-Rad). The blots were striped with stripping buffer (62.5 mM Tris-HCl, 100 mM 2-mercapethanol and 2% SDS at pH 6.7) and re-probed with anti-actin (1:8000) or anti-EGFR (1:2000) antibodies. The band density was quantified by ImageLab version 3.0.1 (BIO-RAD, California, USA).

### Flow cytometry

Seventy percent confluent cells were released from culture flasks with non-enzymatic cell dissociation solution (NECDS, which keep the cell membrane protein intact [33]), washed with PBS and fixed with 2% formaldehyde for 30 min. After two washes with PBS, the cells (5 × 10^5^ cells/ml) were incubated with blocking solution (1% BSA /PBS) for 20 min at room temperate before incubation with 5 µg biotin-galectin-3, 2 µg biotin-PNA, -VVA, -SNA or -GSL-II for 2 h at room temperature. After one wash with PBS and incubation with FITC-avidin (1:500 dilution in 1% BSA) for 1 h. The cells were washed with PBS, resuspend in 1 ml PBS and assessed by flow cytometry (BD FACSCanto, New Jersey, USA) and the data were analysed by Flowing Software 2.5.1 (TURKU Bioscience).

For determination of MGL binding, after incubation with 1% BSA blocking solution, the cells were introduced with 5 µg/ml recombinant MGL in 1% BSA for 1 h at room temperature. After one wash with PBS, the cells were incubated with anti-MGL antibody (2.5 µg/ml in PBS) for 1 h. Following a wash with PBS and incubation with FITC-conjugated secondary antibody (1:200 in PBS) for 1 h, the cells were again washed with PBS, resuspended in 0.5 ml PBS and analysed by Flow cytometry.

### THP-1 monocyte macrophage differentiation

THP-1 cells (5 × 10^5^ cells/ml) were incubated with 10 ng/ml PMA for 48 h at 37 °C for differentiation of the monocytes to M0 macrophages. The culture medium was replaced with fresh medium containing 50 ng/ml IFN-γ and 100 pg/ml LPS for 24 h at 37 °C for differentiation of the cells to M1 macrophages. For differentiation to M2 macrophages, the culture medium of M0 macrophages was replaced with 10 ml fresh medium containing 60 ng/ml IL-4 for 72 h at 37 °C.

For validation of THP-1 differentiation, the cells were released with NECDS, washed once with PBS and fixed with 2% formaldehyde for 30 min. After two washes with PBS, the cells (5 × 10^5^ cells/ml) were incubated with 1% BSA/PBS for 20 min before incubation with PE-conjugated antibodies against M0 marker CD68 [34] (0.25 μg/ml), M1 marker CD86 [35] (0.5 μg/ml), or with PE-conjugated antibody against M2 marker EGR2 [36] (0.25 μg/ml) in PBS for 2 h. After two washes with PBS, the cells were suspended in 1 ml PBS and analysed by Flow cytometry.

### Cancer cell-cell and cancer cell-macrophage interactions

To determine cancer cell–cell interaction, 70–80% confluent cells were released with NECDS, washed with PBS and passed through a 40 μm cell strainer (BD company) to obtain single-cell suspensions. Two 0.5 ml aliquots (5 × 10^5^ cells/ml) of cell suspension were incubated with 8 μl/ml DiO- or DiI-cell labelling solution for 30 min at 37 °C. After washes with PBS, the two cell suspensions were mixed with addition of 5 μg/ml galectin-3 or 5 μg/ml BSA at 37 °C on a rotating platform for 90 min before being immediately analysed by flow cytometry.

To determine cancer cell-macrophage interaction, cancer cells were labelled with DiI-cell labelling solution as described above. Macrophages (10^6^ cells/ml) in 2 ml serum-free medium were incubated with 8 µl DiO for 30 min at 37 °C. After being washed with PBS, the DiO-labelled macrophages were suspended into 5 × 10^5^ cells/ml. Two aliquots of 0.5 ml cell suspension were incubated with 20 μg/ml anti-MGL antibody and one control aliquot of 0.5 ml suspension with PBS for 40 min at room temperature. Half ml DiO-labelled macrophage suspension was then mixed with 0.5 ml DiI-labelled cancer cells (5 × 10^5^ cells/ml) for 1.5 h at 37 °C on a rotation platform before analysed immediately by Flow cytometer.

### EGFR activation

Sixty percent confluent cells were cultured in 12-well plates in serum-free McCoy’s 5 A medium at 37 °C overnight. After two washes with PBS, the cells were incubated with galectin-3 (5 μg/ml) or BSA (5 μg/ml, control) for 30 min before introduction of EGF (20 ng/ml) for 5 min at 37 °C. Cells were washed with ice-cold Tris-buffer (TBS, 50 mM Tris-HCl, pH 7.4) before being lysed with SDS-sample buffer and analysed by immunoblotting.

### Cell proliferation

Sixty percent confluent cells were released from culture flasks by trypsin, washed with PBS, and seeded into 96-well plates at 5 × 10^4^ cell/ml in culture medium. After incubation at 37 °C for various times, cell proliferation was analysed by the CellTiter 96 AQ_ueous_ Non-Radioactive Cell Proliferation kit according to the manufacture’s protocols. The plates were read at 492 nm with reference at 595 nm by a Sunrise^TM^ microplate reader (Tecan, Männedorf, Switzerland).

### Colony formation

Sixty percent confluent cancer cells were released by trypsin, washed once with PBS and passed (5 × 10^4^ cells/ml) though 40 μm cell strainer to obtain single cell suspension. The cells were seeded into 6-well plates (500 cells/well) in culture medium and cultured for eight days at 37 °C (with medium change at day 4). The cells were fixed with 2% paraformaldehyde for 20 min at room temperature and stained with 1% crystal violet solution for 30 min. The cells were washed three times with H_2_O before the number of colonies containing >80 cells were quantified by ImageJ (ImageJ (RRID:SCR_003070, NIH, Maryland, USA).

### Cell migration

Seventy percent confluent cells were released by trypsin, washed with PBS and suspended to 1 × 10^6^ cells/ml. Ninety μl cell suspension was introduced to IBidi 3-well Culture-Inserts in 12-well plates and cultured at 37 °C for 24 h for monolayer formation. The inserts were then removed to create 500 μm gaps and the cells were continued to be cultured. The gaps were monitored by Zeiss Axiovert 25 inverted phase contrast microscope (Zeiss, Jena, Germany) every 24 h.

### Cell adhesion

Twenty-four well culture plates were coated with Matrigel matrix proteins (1:10 dilution with culture medium) for 2 h at 37 °C. Cancer cells were released by trypsin, washed with PBS, resuspended into 5 × 10^4^ cells/ml and incubated with DiO- fluorescence cell labelling solution (5 μl/ml) for 0.5 h at 37 °C. The cells (5 × 10^4^ cells/ml) were washed with PBS and introduced to the matrix-coated plates with addition of 5 µg/ml galectin-3 or 5 µg/ml BSA (control) for 0.5 h. The cells were washed twice with PBS and the number of adherent cells in three (HCT116F3) and ten (SW620) randomly selected fields of views (FOVs) were quantified under fluorescent microscope (Leica, Wetzlar, German) with 40x (SW620) and 10x objectives (HCT116F3).

### Cell viability and proliferation in response to tumour-macrophage interaction

Cancer cells were released from culture flasks by trypsin, washed with PBS and suspended to 10^5^ cells/ml in culture medium containing 10% fatal calf serum. One hundred µl cell suspension was added into the macrophages cultured in 96-well plates for 24 h at 37 °C. Cell viability and cell proliferation were then analysed using the CellTier-Glo^®^Luminescent Cell viability assay kit and CellTier 96^®^Non-Readioactive Cell Proliferation Assay kit, respectively, according to the protocols provided by the kits.

### Macrophage phagocytosis

THP-1 cells were seeded into 24-well plate (4 × 10^5^ cells/well) and differentiated into M1 macrophages as described above. The macrophages were labelled with 8 µl DiO for 40 min at 37 °C. Cancer cells were released by trypsin and washed by PBS and labelled (5 × 10^4^ cells/ml) with 8 µl/ml DiI in culture medium for 40 min at 37 °C. Both cell populations were washed twice by PBS and 1 ml cancer cell suspension (5 × 10^4^ cells) was introduced into macrophage plates for 24 h at 37 °C. The cells were washed twice with PBS and fixed by 2% paraformaldehyde for 20 min at room temperature. The number of phagocytosis in five random selected FOVs were recorded under fluorescent microscope with a 20x objective. Events of green-labelled macrophages overlapped with red-labelled tumour cells (yellow) in single cells were considered as phagocytotic events.

### Cytokine secretion

THP-1 cells were seeded into 12-well plate (8 × 10^5^ cells/well) and differentiated into M1 or M2 microphages as described above before introduction of cancer cells (10^5^ cells/ml) for 24 h at 37 °C. The culture media were collected, centrifuged at 5,000 rpm for 5 min and the concentrations of IL-6 (from cancer-M1 macrophage culture) and IL-10 (from cancer-M2 microphage culture) in the supernatant were measured by IL-6 and IL-10 ELISA. The absorption was measured at 492 nm with reference at 595 nm by Tecan GENios microplate reader.

### Generation of GFP-tagged cells

Lentiviral particles were produced with the transfer vector pLNT-SFFV-EGFP in HEK 293TN cells using two plasmids encoding the viral envelope (pMD2.G) and packaging proteins (psPAX2) (obtained as gifts from Didier Trono and Bryan Welm, Addgene plasmids #12259 and #12260) [37]. SW620shCon and SW620ShG1GT cells were cultured to 50% confluence in 12 well-plates in DMEM culture medium. The cells were transduced with the viral particles (MOI: 10) for 12 h at 37 °C to obtain GFP-tagged cells. The transduction efficacy was above 90% as determined under a fluorescence microscope.

### Tumour growth in Chick embryo

Fertilised eggs were incubated at 37.8 °C in a 45% humidity flow hood (OvaEasy 380 Advance Series II, Brinsea) for three days. The eggs were punctured on the wide end with an egg piercer and 3 ml albumen was removed. A rectangular window (1–2 cm) was cut in the shell to create an area of fenestration. The windows were sealed with adhesive tape and the eggs were returned into the 45% humidified incubator at 37.8 °C for 4 more days. Eight μl SW620shC1GT-GFP or SW620shCon-GFP cell suspension (1.8 × 10^6^ cells) without or with 5 μg galectin-3 was introduced to the egg Chorioallantoic Membrane (CAM). After closure of the windows, the eggs were incubated in the 45% humidified incubator at 37.8 °C for 7 more days. Tumour volumes (Day 14 of Gestation) were measured under a fluorescent microscope (Leica M165 FC with DFC425 C camera) and tumour size was calculated using the equation Volume= Length^2^ x Width /2.

### Generation of C1GaT1 transgenic mice

The mouse model of breast cancer was generated by introducing a specific deficiency of C1galt1 in mammary epithelial cells (PMID:26124270). This was achieved by crossing mice with loxP sites flanking *C1galt1* gene (*C1galt1*^*f/f*^ mice) [38] with a transgenic mouse line expressing Cre recombinase under the control of the mouse mammary tumour virus (MMTV) promoter (PMID: 2898299). The resulting mice with mammary epithelial cell-specific deficiency of C1galt1 (ME *C1galt1*^-/-^) were then bred with MMTV-ErbB2 transgenic mice, which express the activated ErbB2 oncogene under the control of the MMTV promoter (ME *C1galt1*^-/-^/Erb). The control mice were obtained by crossing *C1galt1*^f/f^ mice with MMTV-ErbB2 mice (*C1galt1*^f/f^/Erb). All experiments involving mice were conducted on littermates with a mixed genetic background. The experimental protocols were approved by the Institutional Animal Care and Use Committee of the Oklahoma Medical Research Foundation.

### Immunohistochemistry

Two mice in each group were randomly selected and analysed in this study. The investigator was blinded to the group allocation during the experiment. Paraffin-embedded tumours were sectioned into 4 μm sections by Leica RM2255 microtome (Leica, Wetzlar, German). The sections were rehydrated in H_2_O and treated with 3% H_2_O_2_ for 5 min before incubation with 5% BSA blocking buffer for 30 min. The sections were washed once with TBST buffer and incubated with antibodies against C1GalT1 (BS-9685R, 1:100 dilution), CD11c (1 μg/ml), F4/80 (1 μg/ml), MGL (2.5 μg/ml), or with biotin-PNA (4 μg/ml), or biotin-VVA (4 μg/ml) in 5% BSA at 4 °C overnight. After being washed with TBST, peroxidase-conjugated secondary antibody or avidin-peroxidase were applied for 1 h. The sections were washed by TBS with 0.1% tween 20 and stained with 3,3′-Diaminobenzidine (DAB) Liquid Substrate System tetrahydrochloride (SIGMA) for 8 min. The sections were dehydrated twice by Xylene for 5 min, mounted with DPX mounting medium and imaged with Leica DMLA microscope (Leica, Wetzlar, German). Macrophage and DC infiltration was evaluated by Fiji Image J as previously described [39]. Briefly, colour deconvolution was first applied to the immunohistochemistry images. Each image was then separated into Haematoxylin staining and DAB staining fractions. The DAB fraction was converted into black and white binary images. After background signal removal, the percentage area of signal coverage was measured [39] and was considered as positive infiltration of macrophage and DC.

### Extraction of galectin-3 and MGL binding ligands

SW620shC1GT and SW620shCon cells (8 × 10^6^) were washed twice with PBS and lysed in 2 ml lysis buffer (1% Triton-100, protease inhibitor cocktail in PBS) on a rotating platform for 40 min on ice. After centrifugation at 12,000 rpm for 5 min, the supernatants were collected and three 0.5 ml/cell aliquots were made. One aliquot was introduced with 5 µg recombinant galectin-3, one with 5 µg recombinant MGL and the third one with PBS (control). After incubation for 2 h at room temperature, 5 μg/ml anti-galectin-3 or anti-MGL antibody was added overnight at 4 °C. Protein-A/G agarose beads (100 μl) were washed with lysis buffer before being introduced into each sample for 60 min at room temperature. The beads were collected after centrifugation at 1,000 rpm for 5 min and washed with lysis buffer. Binding proteins were released by incubation of the beads with 60 µl elution buffer (0.1 M glycine at pH 2.5–3.0) for 20 min on a rolling platform. After centrifugation at 5,000 rpm, the supernatants were collected and samples were analysed by mass spectrometer for protein identification.

### Mass spectrometry

The extracted proteins were analysed using an Ultimate 3000 RSLC™ nano-system (Thermo Scientific, Hemel Hempstead) coupled to a Q Exactive™ mass spectrometer (Thermo Scientific). Protein extracts were loaded onto the trapping column (Thermo Scientific, PepMap100, C18, 300 μm X 5 mm), using partial loop injection, for seven min at a flow rate of 12 μL/min with 0.1% Formic Acid (v/v). Sample was resolved on the analytical column (Easy-Spray C18 75 µm x 500 mm 2 µm column) using a gradient of 96.2% A (0.1% formic acid) 3.8% B (79.95% acetonitrile, 19.95% water, 0.1% formic acid) to 50% A 50% B over 45 min at a flow rate of 0.3 nL/min (1-hour programme). The data-dependent programme used for data acquisition consisted of a 70,000-resolution full-scan MS scan in the orbitrap (AGC set to 1e6 ions with a maximum fill time of 250 ms). The 10 most abundant peaks per full scan were selected for HCD MS/MS (35,000 resolution, AGC set to 1e5 ions with a maximum fill time of 100 ms) with an ion selection window of 2 m/z and normalised collision energy of 30%. Ion selection excluded singularly charged ions and ions with equal to or a greater than +5 charge state. To avoid repeated selection of peptides for fragmentation the programme used a 20-second dynamic exclusion window. All the samples, each from three independent extractions, were analysed, each in triplicate, in random order with blanks between each condition.

### Statistical analysis

One-way analysis of variance (ANOVA) followed by Bonferroni correction was used for multiple comparisons and two tailed independent t-test was used for comparison of two groups using SPSS 26. Difference was determined when *p* < 0.05.

## Results

### C1GalT1 suppression reduces TF and increases Tn expression in human colon cancer cells

**Fig. 1:**
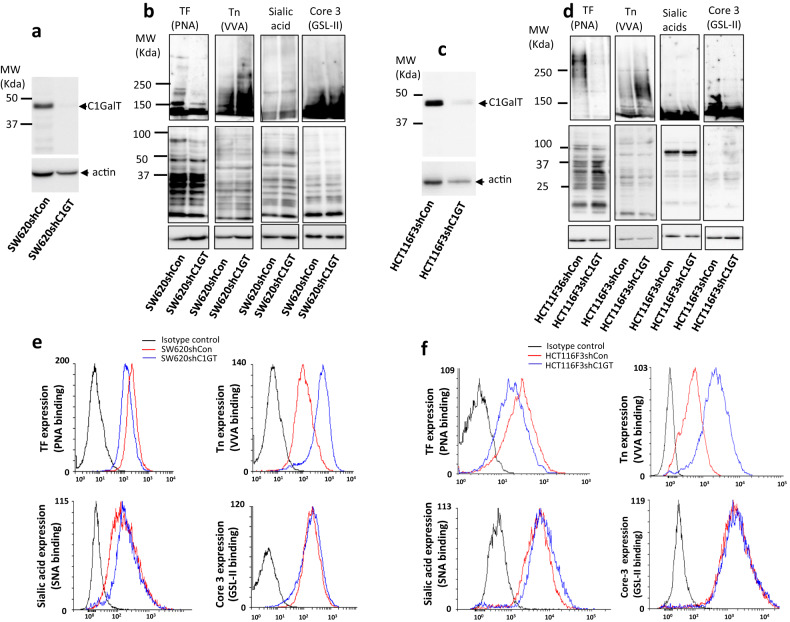
shRNA C1GalT1 suppression reduces TF and increases Tn expression but has little influence on expressions of terminal sialic acid and Core 3 carbohydrate structures in human colon cancer cells. shRNA C1GalT1 transfection led to substantial reduction of C1GalT1 in SW620 (**a**) and HC116F3 (**c**) cells assessed by C1GalT1 immunoblotting. **b**, **d** shRNA C1GalT1 suppression led to reduction of TF and increase of Tn structures of cellular proteins of SW620 (**b**) and HCT116F3 (**d**) cells when analysed by lectin blotting using TF-binding PNA and Tn-binding VVA. No change of terminal sialic acids (SNA binding) and Core 3 (GSL-II) structures occurs in control and C1GalT1 suppressed cells. **e**, **f** Analysis of cell surface expressions of TF, Tn, terminal sialic acid and Core 3 structures by lectin blot and flow cytometry and show similar results as by lectin blots of SW620 (**e**) and HCT116F3 (**f**) cells. Representative blots and flow cytometry histograms from two independent experiments are shown.

To determine the impact of C1GalT1 expression on protein O-glycosylation, human colon cancer SW620 and HCT116F3 cells were transfected with C1GalT1 shRNA or control shRNA to generate stable C1GalT1 knockdown cells. shRNA transfection resulted in 93% (Fig. 1a) and 73% (Fig. 1c) reduction of C1GalT1 expression in SW620 and HCT116F3 cells, respectively. C1GalT1 knockdown decreased TF and increased Tn expression of a number of cellular proteins, in particular higher molecule weight proteins, when assessed by lectin blotting using TF-binding peanut agglutinin (PNA) and Tn-binding VVA in both SW620 (Fig. 1b) and HCT116F3 (Fig. 1d) cells. No obvious changes of terminal sialic acids and Core 3 carbohydrate structures were seen between C1GalT1-kockdown and control cells when assessed by sialic acid-binding lectin SNA and terminal N-acetylglucosamine-binding lectin GSL-II. Similar results were observed on cell surface proteins when lectin cell surface binding was analysed by flow cytometry (Fig. 1e, f). A 35.8 ± 12.2% (mean ± SD) and 37.0.6 ± 3.9% reduction of TF expression, 587.6 ± 252.1% and 352.4 ± 69.5% increase of Tn expression was seen in SW620shC1GT (Fig. 1e) and HCT116F3shC1GT (Fig. 1f) cells in comparison to the control cells. Changes of cell surface sialic acid and Core 3 structures, like that shown in total cellular proteins (Fig. 1b, d), were again minimal in both SW620 and HCT116F3 cells in response to C1GalT1 suppression. These results indicate that reduction of C1GalT1 expression decreases the expression of TF and increases the expression of Tn carbohydrate structures. It also indicates that C1GalT1 suppression has minimum influence on the expressions of Core 3 structure and terminal sialic acids in those cells.

### Change of C1GalT expression alters tumour cell behaviours

**Fig. 2:**
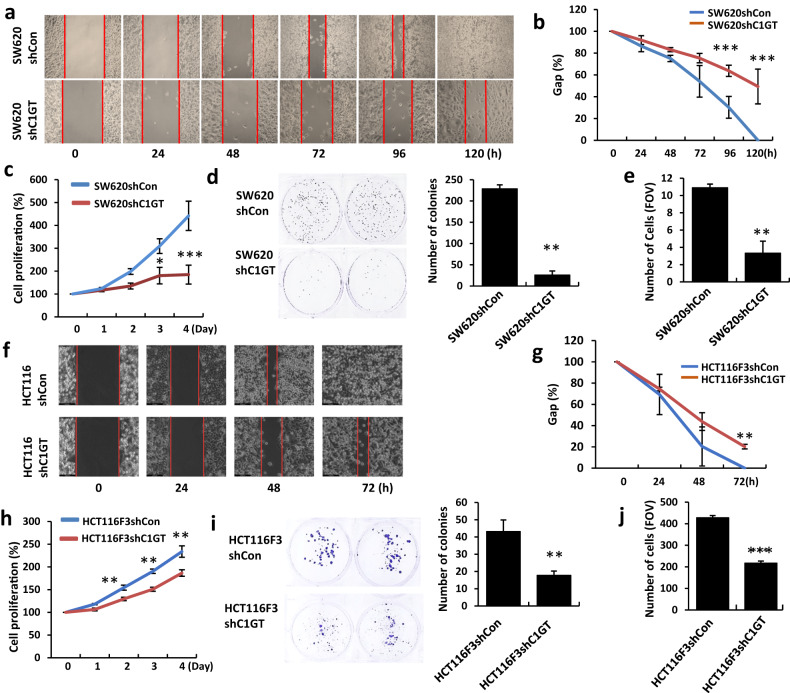
C1GalT suppression alters cancer cell activities. C1GalT1 suppression reduces migration of SW620 (**a**, **b**) and HCT116F3 (**f**, **g**) cells. The distance between the two-cell migrating fronts was quantified and is shown as percentage of 0 h (**b**, **g**). C1GalT1 suppression in SW620 (**c**) and HCT116F3 (**h**) cells leads to reduction of cell proliferation and colony formation of SW620 (**d**) and HCT116F3 (**i**) cells. The bar charts show average number of colonies with >80 cells. C1GalT1 suppression reduces adhesion of SW620 (**e**) and HCT116F3 (**j**) cells to matrix proteins. Number of adherent cells from at least 10 randomly selected FOVs for SW620 and three for HCT116F3 cells were quantified. Data are expressed as mean ± SEM from three independent experiment, each in triplicate, *p < 0.05, **p < 0.01, ***p < 0.001 (ANOVA followed by Bonferroni).

In comparison to control cells, C1GalT1 suppression significantly reduced the ability of both SW620 and HCT116F3 cells in migratione (Fig. 2a, f), proliferatione (Fig. 2c, h), adhesion (Fig. 2e, j) and colony formation (Fig. 2d, i). While the control cells completed migration across the 500 µm gaps within 120 and 72 h, respectively, 50% and 20% gaps remained at 120 and 72 h in SW620shC1GT (Fig. 2a, b) and HCT116F3shC1GT (Fig. 2f, g) cells. At day 4, proliferation of SW620shC1GT and HCT116shC1GT cells were 257% and 47% lower than SW620shCon and HCT116shCon cells, respectively. The average number of colonies formed by C1GalT1 knockdown cells were 9- and 2-fold less, respectively, than the SW620shCon (Fig. 2d) and HCT116F3shCon (Fig. 2i) cells after 7-day culture. The number of adherent cells following C1GalT1 suppression was 4- and 2-fold lower, respectively, than the control SW620 (Fig. 2e) and HCT116F3 (Fig. 2j) cells. These results indicate that reduction of C1GalT1 expression substantially reduces tumour cell activities that are crucial in cancer development and progression.

### Suppression of C1GalT expression reduces galectin-3-mediated tumour cell activities

**Fig. 3:**
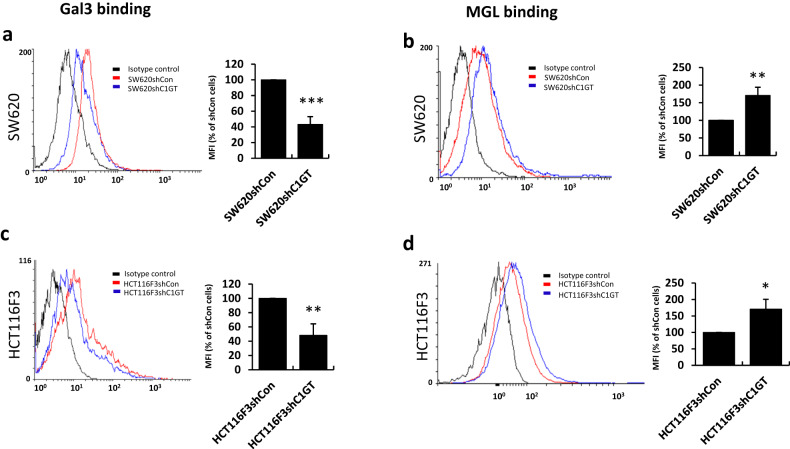
C1GalT suppression reduces galectin-3 binding but increases MGL binding to human colon cancer cells. Binding of galectin-3 (Gal3) (**a**, **c**) and MGL (**b**, **d**) to SW620shCon and SW620shC1GT cells (**a**, **b**) and HCT116F3shCon and HCT116F3shC1GT (**c**, **d**) was assessed by flow cytometry using biotinylated galectin-3 or MGL followed by anti-MGL antibody and fluorescence-conjugated secondary antibody. Representative flow cytometry histograms are shown. Percentage changes of the mean fluorescent intensity (MFI) (% of shCon cells), shown in the right on each histogram, are expressed as mean ± SD from three independent experiment. *p < 0.05, **p < 0.01, ***p < 0.001 (T-test).

As TF and Tn antigens are each natural binding ligands of galectin-3 and MGL, we then assessed the effect of C1GalT1 change on binding of galectin-3 and MGL to the cells. It was found that suppression of C1GalT1 expression caused a reduction of 57% and 52%, respectively, of galectin-3 binding to SW620 and HCT116F3 cells (Fig. 3a, c). On the other hand, C1GalT1 suppression resulted in an increase of 71% and 70%, respectively of MGL binding to SW620 and HCT116F3 (Fig. 3b, d). These suggest that change of C1GalT1 expression in tumour cells can substantially alter tumour cell interaction with galectin-3 and MGL.

**Fig. 4:**
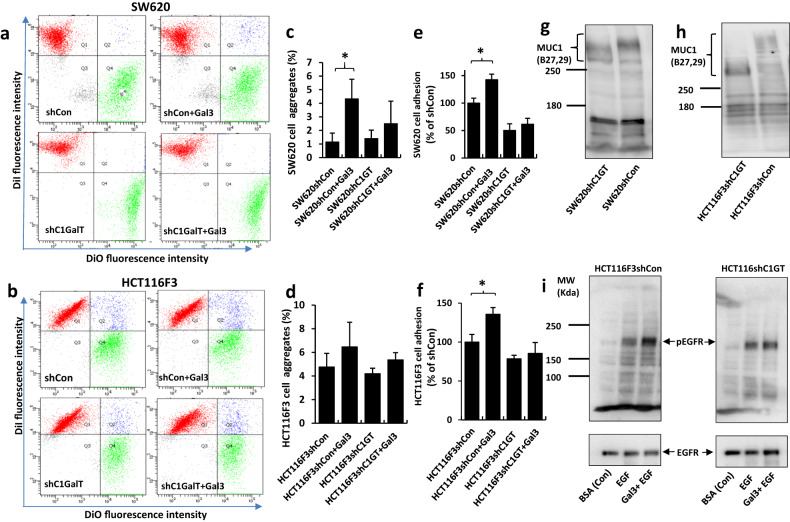
C1GalT1 suppression reduces galectin-3-mediated tumour cell-cell interaction and cell adhesion to basement proteins. **a**, **b** Equal number (5 × 10^5^ cells/ml) of DiO- and Dil-labelled SW620shCon or SW620shC1GalT1 (**a**); HCT116F3shCon or HCT116F3shC1GalT1 (**b**) cells were mixed without or with 5ug/ml recombinant galectin-3 for 90 min before analysis by flow cytometry. Cell population occurs at the top right (blue) in the bivariate correlation plot which contains both DiO- and Dil-labled cells are defined in this study as cell-cell aggregates/interaction. Representative flow cytometry plots are shown in (**a**) and (**b)** . Percentage changes of cell aggregates of shC1GT in comparison to shCon cells are expressed as mean ± SD from four (**c**, SW620) and three (**d**, HCT116F3) independent experiments. *p < 0.05 (T-test). **e**, **f** C1GalT suppression reduces galectin-3-mediated tumour cell adhesion to matrix proteins. Percentage adhesion of shC1GT cells in comparison to shCon cells are shown in **e** (SW620) and **f** (HCT116F3). Data are expressed as mean ± SEM of three independent experiments, each in triplicate *p < 0.05 (ANOVA, followed by Bonferroni). **g**, **h** MUC1 immunoblots show reduction of MUC1 molecular size in HCT116F3shC1GT (**g**) and SW620sgC1GT (**h**) cells in comparison to control cells. **i** C1GalT1 suppression reduces galectin-3-mediated EGFR phosphorylation in cell response to EGF in HCT116F3 cells. Representative blots from three independent experiments are shown.

Previous studies have reported that binding of galectin-3 to TF on the transmembrane mucin protein MUC1 [32] of tumour cells induces tumour cell-cell homotypic aggregation that promotes circulating tumour cell resistance to anoikis in hematogenous dissemination [33]. Galectin-3 is also known to enhance tumour cell adhesion to basement proteins by interaction with cell surface or matrix glycans in cancer progression [40]. To determine the impact of C1GalT1 change on galectin-3-mediated tumour cell behaviours, SW620 and HCT116F3 cell-cell interaction and cell adhesion were assessed in the presence of galectin-3 at a pathological concentration similar to that seen in colorectal cancer patients with metastasis [41]. The presence of galectin-3 led to 258% (from 1.2% to 4.3%) and 36% (from 4.8% to 6.5%) increase, respectively, of cell-cell aggregation of SW620shCon (Fig. 4a, c) and HCT116F3shCon (Fig. 4b, d) in comparison to the cells without galectin-3. This effect of galectin-3 was reduced to 79% (1.4% to 2.5%) and 28% (from 4.2% to 5.4%), respectively, in SW620shC1GT and HCT116F3shC1GT cells. The presence of galectin-3 also led to a significant increase (43% and 36% respectively) of SW620shCon (Fig. 4e) and HCT116F3shCon (Fig. 4f) cell adhesion to basement proteins but this effect of galectin-3 was completely lost in C1GalT1 knockdown cells.

Our early study has demonstrated that binding of galectin-3 to TF on MUC1 enhances EGF-induced EGFR activation by promoting MUC1-EGFR interaction and EGFR dimerization [32]. It was found here that C1GalT1 suppression in SW620 and HCT116F3 cells substantially reduced MUC1 O-glycosylation as demonstrated by reduction of MUC1 molecular weight in SDS-PAGE (Fig. 4g, h). Introduction of galectin-3 led to 67.3 ± 10.1% increase of EGF-induced EGFR phosphorylation in HCT116F3shCon cells, as reported before [32], but not in HCT116F3shC1GT cells (Fig. 4i). These results indicate that suppression of C1GalT1 expression in cancer cells significantly reduces galectin-3-mediated tumour cell-cell interaction, adhesion and EGFR activation.

### Suppression of C1GalT expression increases MGL-mediated macrophage-tumour cell interaction

MGL is known to be expressed predominately by macrophages and DCs [42, 43]. Having shown that suppression of C1GalT1 increases MGL binding to tumour cells (Fig. 1 and Fig. 3), we then investigated the influence of C1GalT1 alteration on tumour cell interaction with macrophages differentiated from THP-1 monocytes. THP-1 monocytes were differentiated into M0, M1 and M2 macrophages by PMA (M0), IFN-γ and LPS (M1) and IL-4 (M2) [36]. After PMA treatment, 94% cells showed expression of macrophage marker CD68 while 9% and 3% expression of CD86 and EGR2, indicating well differentiation of the THP-1 cells to M0 macrophages (Fig S1). Treatment of the PMA-differentiated macrophages with IFN-γ and LPS resulted in 93% expression of M1 macrophage marker CD86 while no expression of M2 marker EGR2. While treatment of the PMA-differentiated macrophages with IL-4 led to 94% expression of M2 macrophage marker EGR2 but no expression of M1 macrophage marker CD86. These results indicate PMA-differentiated M0 macrophages treated further with IFN-γ and LPS or with IL-4 were predominately M1 and M2 macrophages respectively.

**Fig. 5:**
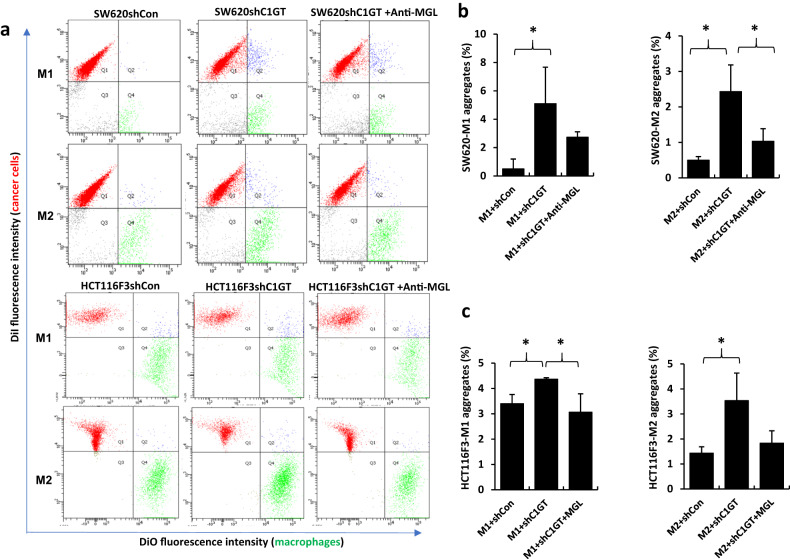
C1GalT suppression increases MGL-mediated macrophage-tumour cell interaction. Equal numbers (5 × 10^5^ cells/ml) of Dil-labelled cancer cells and DiO-labelled macrophages were mixed in the absence or presence of anti-MGL antibody (20 μg/ml) for 40 min before analysis by flow cytometry. The top right (blue) in each of the bivariate correlation plots that contain both DiO-labelled macrophages and Dil-labelled cancer cells are defined as tumour-macrophage aggregates/interaction. Representative flow cytometry plots from two independent experiments are shown in (**a**) (numbers in the correlation plots show percentage cell population). Percentage changes of M1 and M2 macrophages-tumour cell aggregates of shC1alT1 cells, without or with anti-MGL antibody, in comparison to shCon cells are shown in (**b**) (SW620) and (**c**) (HCT116F3). Data are expressed as mean ± SD from three independent experiments. *p < 0.05 (T test).

It was found that when macrophages were introduced with shC1GalT1 and shCon tumour cells, macrophage interaction with shC1GT cells (represented by the formation of macrophage-tumour cell heterotypic aggregates) was substantially higher than with shCon of both SW620 and HCT116F3 cells (Fig. 5a). The number of heterotypic aggregates formed by M1 and M2 macrophages with SW620shC1GT cells were 10.2- and 4.8-fold higher, respectively, than with SW620shCon cells (Fig. 5a, b). The number of heterotypic aggregates formed by M1 and M2 macrophages with HCT116F3shC1GT cells were 1.3- and 2.5-fold higher, respectively, than with the HCT116F3shCon cells (Fig. 5a, c). Furthermore, introduction of an anti-MGL antibody caused a reduction of 46% and 56% respectively, of the increase of macrophage-SW620shC1GT cell aggregation with M1 and M2 types of macrophages (Fig. 5a, b). Similarly, the presence of anti-MGL antibody led to an inhibition of 30% and 48% respectively, in the increase of macrophage- HCT116F3shC1GT cell aggregation with M1 and M2 macrophages (Fig. 5a, c). Together, these results indicate that lower C1GalT1 expression by tumour cells lead to greater interaction of the tumour cells with macrophages and this interaction is largely mediated by MGL.

### C1GalT1-associated change of tumour-macrophage interaction alters macrophage action in cytokine secretion

Macrophages are one of the most abundant population of immune cells in the tumour microenvironment of solid tumour. Tumour-associated macrophages (TAM) are heterogenic and can be either pro-inflammatory (M1) and anti-inflammatory (M2) in response to microenvironmental perturbation [44]. M1 macrophages generally contribute to tumour cell destruction by either engulfing or by releasing cytotoxic factors [44] and pro-inflammatory cytokines IL-6, IL-12 and TNF-α [44]. M2 microphages on the other hand typically aid cancer progression by secreting anti-inflammatory cytokines such as IL-10 to inhibit DC maturation and antigen presentation, and help T regulator (T_Reg_) cell recruitment to the tumour microenvironment to exert immunosuppression [45, 46]. M2 macrophages also produce EGF [47] and vascular endothelial growth factor A (VEGFA) to promote tumour growth and angiogenesis [48].

**Fig. 6:**
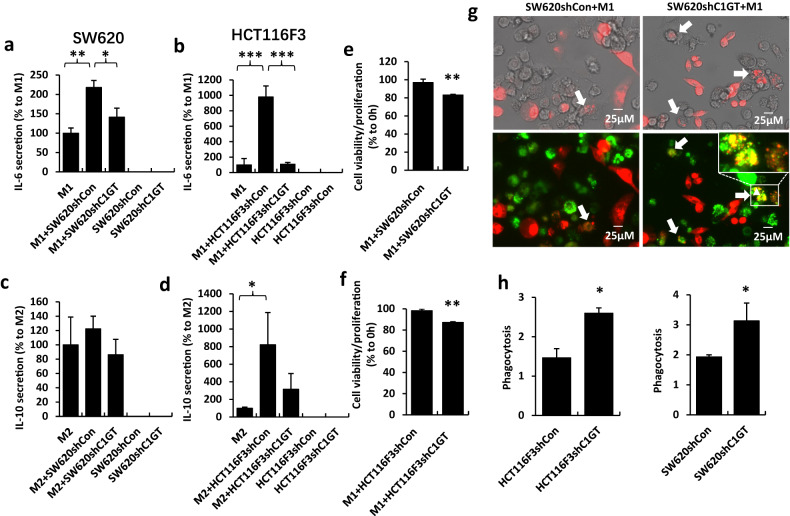
Macrophages secrete higher levels of cytokines in interaction with C1GalT1 suppressed tumour cells than with shCon cells. Macrophage interaction with SW620shC1GT and HCT116F3shC1GT tumour cells leads to higher macrophage secretion of IL-6 and IL-10 in comparison to interaction with shCon cells. SW620 (**a**) or HCT116F3 (**b**) cells were introduced to M1 (**a**, **b**) or M2 (**c**, **d**) macrophages for 90 min and the concentrations of IL-6 (**a**, **b**) or IL-10 (**c** and **d**) in the culture medium were analysed by ELISAs. Data are expressed as mean ± SEM of three independent experiment, each in triplicate. **e**, **f**, shC1alT1 tumour cells show reduced viability than shCon cells after interaction with M1 macrophages. M1 macrophages were incubated with SW620 (**e**) or HCT116F3 (**f**) cells for 24 h before cell proliferation as well as cell viability were separately analysed. Data are expressed as mean ± SEM of thee independent experiments, each in triplicate. Percentage cell viability/proliferation of shC1GT cells in compassion to shCon cells are shown in (**e**) and (**f**). *p < 0.05, **p < 0.01, ***p < 0.001 (ANOVA, followed by Bonferroni). **g** More macrophage-tumour phagocytosis is seen in M1 macrophage (DiO labelling, green) interaction with shC1GT cells (DiI labelling, red) than with shCon cancer cells (DiI labelling, red). Representative images are shown (arrows point to phagocytotic events). **h** Number of phagocytotic events in five randomly selected FOVs were quantified and data are shown as mean ± SEM from three independent experiment, *p < 0.05 (T test).

Having shown that C1GalT1 suppression in tumour cells increases tumour cell interaction with macrophages, we then assessed the influence of such interaction on macrophage activities. Interaction of M1 macrophages with SW620shCon (Fig. 6a) and HCT116F3shCon (Fig. 6b) cells was seen to lead to an increase of 118% and 879%, respectively, of IL-6 secretion in comparison to resting M1 macrophages. Interaction of the M1 macrophages with C1GalT1 knockdown cells also increased IL-6 secretion but to a much lower level (41% and 8% respectively) (Fig. 6a, b). Interaction of M2 macrophages with HCT116F3shCon cells led to a 721% increase of IL-10 secretion while interaction with HCT116F3shC1GT cells increased by 215% (Fig. 6d). Interaction of M2 macrophages with SW620 cells did not show a significant effect on IL-10 secretion with or without C1GalT1 suppression (Fig. 6c). Secretion of IL-6 and IL-10 by SW620 and HCT116F3 cells were both undetectable (Fig. 6a–d), indicating the observed increase of IL-6 and IL-10 secretion in macrophage-tumour interaction came predominately from the macrophages. This suggests that macrophages, in particular M1 type macrophages, secret significantly higher amounts of cytokines when they interact with C1GalT1-expressing tumour cells than with C1GalT1-suppressed tumour cells.

In addition to the release of cytotoxic molecules and pro-inflammatory cytokines to exert their anti-cancer effects, M1 macrophages can also destroy tumour cells directly by phagocytosis [44]. Tumour cells labelled by DiI (red) overlapped with macrophages labelled by DiO (green) are counted as phagocytotic events. Interaction of M1 macrophages with C1GalT1 knockdown SW620shC1GT and HCT116F3shC1GT cancer cells was found to be associated with 62%% and 78%, respectively, higher macrophage-tumour cell phagocytotic events in comparison to interaction with SW620shCon and HCT116F3shCon cells (Fig. 6g, h). When the cell viability as well cell proliferation was measured directly, interaction of SW620shC1GT (Fig. 6e) and HCT116F3shC1GT (Fig. 6f) cells with M1 macrophages was seen to be associated with a 13% and 11%, respectively, lower cell viability in comparison to interaction of the control cells with M1 macrophages. These results suggest that higher C1GalT1 expression by tumour cells favours survival of the tumour cells in the tumour microenvironment as a result of reduced tumour cell interaction with tumour-destructive M1 macrophages.

### Reduction of C1GalT expression is associated with slower tumour growth in vivo

**Fig. 7:**
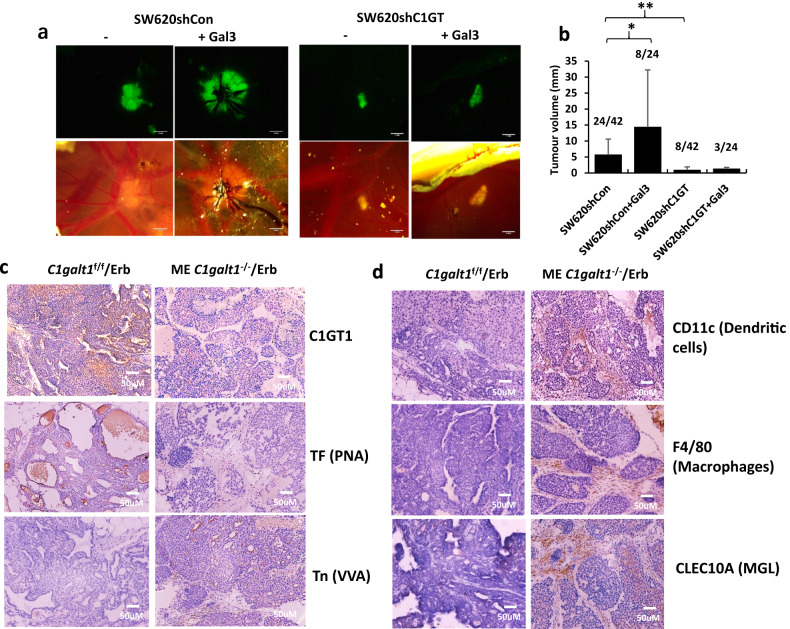
C1GalT suppression reduces tumour growth in chicken embryos and more macrophages and dendritic cells are accumulated in the tumour microenvironment of ME *C1galt1*^-/-^/Erb mice than of *C1galt1*^f/f^/Erb mice. GFP**-**SW620shC1GT and GFP-SW620shCon cells (1.8 × 106 cells) without or with 5 µg galectin-3 was implanted in the CAMs at Day 7 and tumour sizes were measured at Day 14 of embryonation. Representative images of the tumours grown on CAM at Day 14 are shown in (**a**). Tumour volume are shown in (**b**) (number of eggs with detectable tumour/total number of eggs at Day 14 are also shown on top of each bar). Data are expressed as mean ± SD, *p < 0.05, **p < 0.01 (T test). **c**, **d** Fewer macrophages and dendritic cells were seen in the tumour microenvironment of *C1galt1*^f/f^/Erb mice than in ME *C1galt1*^-/-^/Erb mice. Lower expression of TF and higher expression of Tn were found in the tumours of ME *C1galt1*^-/-^/Erb mice compared with *C1galt1*^f/f^/Erb mice. Higher number of macrophages (F4/80 positive, MGL-positive) and dendritic cells (CD11c positive, MGL positive) are seen in the tumours of ME *C1galt1*^-/-^/Erb mice compared with *C1galt1*^f/f^/Erb mice. Two mice in each group were randomly selected and analyzed and representative images are shown.

The chicken embryo is rich of capillary network and is increasingly used as an effective in vivo model in assessing tumour growth [37] and was used here to assess the effects of C1GalT1 expression on tumour growth without and with galectin-3. It was found that in comparison to implantation with SW620shCon cells in which 57% eggs (24/42) formed tumours, only 19% eggs (8/42, *p* < 0.001) formed detectable tumours when implanted with SW620shC1GT cells (Supplement Table S1). The average tumour size formed by SW620shC1GT cells was also significantly smaller than that formed by SW620shCon cells (Fig. 7a, b). The presence of galectin-3 did not show to have significant effect on the number of eggs forming detectable tumour (Table S1). However, its presence caused a 2.5-fold increase of the tumour size in the SW620shCon cells (*p* = 0.036) but not in the SW620shC1GT cells (Fig. 7b). These results indicate that higher C1GalT1 expression in tumour cells is associated with higher tumour growth in vivo and the presence of galectin-3 has a positive influence on this process.

### Higher C1GalT1 expression by tumour cells is associated with fewer macrophages and DCs in the tumour microenvironment in C1GalT1 transgenic mice

Having shown that change of C1GalT1 expression alters tumour cell interaction with macrophages, mediated by MGL in vitro, we then investigated whether change of C1GalT expression is associated with change of macrophage activity in the tumour microenvironment in a mouse model in vivo [38].

Consistent with our in vitro discoveries, tumours formed in the *C1galt1*^f/f^/Erb mice were seen to express higher amounts of TF (12.8 ± 4.8% staining) and low levels of Tn (<1% staining) structures than those in ME *C1galt1*^-/-^/Erb mice (Fig. 7c, Fig. S2). A higher level of MGL expression was observed within tumours as well as in the tumour surroundings in ME *C1galt1*^-/-^/Erb mice (12.7 ± 4.3% staining) than in *C1galt1*^f/f^/Erb mice (3.2 ± 0.5%) (Fig. 7d). Substantially more macrophages (as illustrated by positive staining of macrophage marker F4/80 and MGL) and dendritic cells (as shown by positive staining of DC marker CD11c and MGL) were observed in the tumour surroundings in ME *C1galt1*^-/-^/Erb mice (7.2 ± 2.0% F4/80 staining and 6.4 ± 2.0% CD11c staining) than in *C1galt1*^f/f^/Erb mice (1.2 ± 0.01% F4/80 staining and 1.3 ± 0.01% CD11c staining) (Fig. 7d). This indicates that tumours with lower C1GalT1 expression attract more MGL-expressing macrophages and dendritic cells into the tumour microenvironment.

### Change of C1GalT1 expression alters the bindings of galectin-3 and MGL to cell surface glycans

To gain further insight into the influence of C1GalT1 change on tumour cell activities mediated by galectin-3 and MGL, we further analysed the cell surface binding receptors of galectin-3 and MGL in response to change of C1GalT expression in cancer cells. A total of 53 cell membrane proteins were extracted by galectin-3 from SW620 cells (Table 1 and Table S2). Twenty fewer cell membrane proteins were extracted by galectin-3 from SW620shC1GT than from SW620shCon cells (Table 1). Well-known galectin-3-binding cell membrane glycoproteins such as MUC1 [32] and integrin [49] were extracted only from SW620shCon cells but not from SW620shC1GT cells. This indicates that suppression of C1GalT1 in cancer cells substantially reduced galectin-3 binding to its cell membrane receptors. For MGL- binding, a total of 34 cell membrane proteins were extracted by MGL from SW620shCon cells, of which 9 were not pulled out from SW620shC1GT cells (Table 1 and S2). Ten additional cell membrane proteins however were extracted by MGL from SW620shC1GT cell (Table 1). Known MGL-binding proteins such as Ep-CAM and macrophage migration inhibitory factor (MIF) were extracted by MGL only from SW620shCon but not from SW620shC1GT cells. These results indicate that, in contrast to a general reduction of galectin-3 binding to its cell surface receptors following C1GalT1 suppression, MGL binding to its cell surface receptors are generally increased in response to reduction of C1GalT1 expression in tumour cells.

**Table 1:** Cell membrane glycoproteins extracted by galectin-3 and MGL differentially from SW620shCon and SW620shC1GT cells.

Galectin-3 extraction		MGL extraction	
Extracted only from SW620shCon cells	Extracted only from SW620shC1GalT1 cells	Extracted only from SW620shCon cells	Extracted only from SW620shC1GalT1 cells
14-3-3 protein sigma	Calcium and integrin-binding protein 1	Catenin beta-1	NMT 1
AP-2 complex subunit alpha-1	CD63 antigen	CD71 antigen	CK II beta
Catenin beta-1	CLEC10A	Ep-CAM	DnaJ protein SB73
CD71 antigen	Interferon-induced transmembrane protein 3	Galectin-8	Glycogen phosphorylase
Copine-1	Myoferlin	Macrophage migration inhibitory factor	Melanoma-associated antigen D2
Desmoplakin	VTI1B	PA28beta	Myoferlin
Galectin-8		PP2A subunit A isoform	Protein cornichon homologue 4
Integrin alpha-6		Sodium pump subunit alpha-1	Protein S100-A10
LETM1 domain-containing protein 1		AP-2 complex subunit beta	Protein S100-P
Macrophage migration inhibitory factor			Uncharacterised protein C7orf50
MagT1			
MCT 1			
MUC-1			
Multidrug resistance-associated protein 1			
P56			
Protein BSCv			
Protein FAM38A			
Protein S100-A4			
Sideroflexin-1			
Spectrin beta chain, non-erythrocytic 2			
Transmembrane protein 109			
ZO-1			
Anion exchange protein 2			
OCIA domain-containing protein 1			
Podocalyxin			
Ragulator complex protein LAMTOR3			

## Discussion

This study demonstrated that change of C1GalT1 expression by shRNA in colon cancer cells substantially alters the O-glycosylation profile of cellular proteins. This includes the reduction of the oncofetal TF and increase of Tn carbohydrate antigens. These glycosylation changes reduce galectin-3-mediated tumour cell-cell interaction, cell adhesion and EGFR activation and decrease galectin-3-mediated tumour growth in a chick embryo model. On the other hand, the changes of cell glycosylation in response to C1GaT1 suppression significantly increase MGL binding, MGL-mediated macrophage-tumour interaction and macrophage phagocytosis and cytokine secretion. Tumours in ME *C1galt1*^-/-^/Erb mice was shown to attract more MGL-expressing macrophages/dendritic cells in the tumour microenvironment than tumours in *C1galt1*^f/f^ /Erb mice.

It has been reported previously that C1GalT1 is overexpressed in 67.5% of colorectal cancer patients. While all patients with low C1GalT1 expression survived over 2000 days, this was reduced to 20% in the patients with higher C1GalT1 expression [21]. Suppression of C1GalT1 by shRNA was shown in this study to result in a substantial reduction of TF and a significant increase of Tn structures of cellular glycoproteins. These glycosylation changes are consistent with the action of C1GalT1 in the bio-synthesis of O-linked glycosylation [4]. Interestingly, suppression of C1GalT1 expression did not seem to affect the appearance of terminal sialic acids and Core 3 carbohydrate structures (Fig. 1). This indicates that although the initial GalNAc residue in O-glycosylation biosynthesis is competed for by three types of glycosyltransferases (C1GalT1, β3GnT, and ST6GalNAc-transferase) [4], C1GalT1 is probably the predominate force, at least in these cancer cells.

Change of protein glycosylation occurs in all cancer stages of every cancer type [17]. Although change of O-linked mucin type glycans is one of the most common glycosylation changes in epithelial cancer [17], our understanding of the functional importance of these glycosylation changes is still in infancy. Many of the glycosylation changes in cancer are known to lead to the appearance of short oncofetal carbohydrate structures such as TF, Tn and sialyl-Tn antigens [50]. These short chain carbohydrate antigens occurs in more than 90% of tumours of epithelial origin but are rarely seen in normal tissues [9] and their appearance is often associated with metastasis and poor prognosis [50]. C1GalT1 is the sole glycosyltransferase in O-glycosylation biosynthesis machinery for the formation of Core 1-associated carbohydrate structures and is broadly overexpressed in epithelial cancers [6]. Overexpression of C1GalT1 is consistently reported to be associated with tumour malignancy, poor prognosis and poor patients’ survival [21–23]. In this study, suppression of C1GalT1 expression in human colon cancer cells is shown to cause significant reduction of tumour cell proliferation, migration, adhesion and colony formation. This supports a positive role of C1GalT1 overexpression in promotion of cancer progression [22, 23]. This perhaps is not surprising as most cell membrane proteins are glycoproteins. As C1GalT1 controls a core O-glycosylation biosynthesis pathway, change of C1GalT1 likely alters the glycosylation status of many cell membrane proteins that are critical in tumour cell activities. Indeed, many cell membrane glycoproteins, for example integrins and MUC1 which are known to be involved in cancer progression, showed glycosylation changes in response to C1GalT1 suppression in this study. Integrin-α6, which was pulled out by galectin-3 only from control but not C1GalT1 suppressed cells in this study (Table 1), is known to carry multiple N- and O-linked glycans on its extracellular domain [51] and is known to be directly involved in epithelial cancer cell adhesion, migration, angiogenesis and invasion [51]. Although it is not yet known how the O-glycans on these cell membrane molecules such as that on integrins are involved in the regulation of these tumour cell activities, some of their influences likely involves their interaction with galectins. An early study showing that galectin-3 interaction with integrin-β3 increases melanoma cell adhesion and metastasis [52] is in keeping with this possibility.

One of the exciting discoveries in this study is the revelation that suppression of C1GalT1 expression reduces the tumour cell interaction mediated by galectin-3 but reciprocally increases their interaction with macrophages mediated by MGL. Galectin-3 is a multi-mode promoter in cancer development, progression and metastasis [53]. It is commonly overexpressed in most types of cancers [54] and promotes multiple steps in cancer progression and metastasis by interaction with galactose-terminated cell membrane glycoproteins [54, 55]. For example, binding of galectin-3 to TF on cancer-associated transmembrane mucin protein MUC1 increases cancer cell heterotypic adhesion to vascular endothelium [56] and promotes endothelial secretion of metastasis-promoting cytokines such as GM-CSF and IL-6 [41, 56]. Binding of galectin-3 to TF/MUC1 on cancer cells also increases cancer cell-cell homotypic aggregation and survival in the blood circulation [33]. Binding of galectin-3 to MUC1 also enhances EGF-induced EGFR activation in epithelial cancer cells by increasing MUC1-EGFR interaction [32, 56]. In this study, the galectin-3-mediated tumour cell-cell homotypic aggregation and promotion of EGF-induced EGFR activation are seen to be significantly reduced following C1GalT1-suppression. This not only provides further support to the action of galectin-3-TF/MUC1 interaction in tumour cell-cell homotypic interactions, it also highlights an important role of cell glycosylation in tumour cell communication with adjacent cells in the tumour microenvironment.

MGL is the sole C-type lectin that binds GalNAc (Tn) in the immune system. It is predominately expressed by macrophages and DCs [12]. Macrophages are the most abundant population of immune cells in the tumour microenvironment [44]. TAMs can either promotes (M2 type) or reduce (M1 type) cancer progression through their immune reactions [48]. M1 type macrophages can exert their immune influence on tumour cells either directly by phagocytosis or indirectly by secretion of cytotoxic factors [44] or pro-inflammatory cytokines [44]. In this study, interaction of M1 macrophages with C1GalT1-suppressed tumour cells is associated not only with higher tumour cell engulfing but also with lower macrophage secretion of IL-6 in comparison to their interaction with higher C1GalT1-expressing cells. As a pleiotropic cytokine, IL-6 plays diverse roles in inflammation and cell growth [57] by binding to its cell surface receptor IL-6Rα [58]. Thus, lower IL-6 secretion by TAMs as a result of their interaction with lower C1GalT1 expressing tumour cells will likely have a less positive influence on tumour growth in the TME.

M2 type macrophages generally promote tumour progression indirectly by secretion of growth factors and anti-inflammatory cytokines such as IL-10 [59]. As an anti-inflammatory, pleiotropic cytokine [60], IL-10 can contribute to cancer progression through several ways by binding to one of its two glycosylated receptors IL-10R1 on T cells, B cells, natural killer cells, mast cells, monocytes or DCs, to inhibit inflammation [61].or by binding to its alternative receptor IL-10R2 to inhibit production of pro-inflammatory cytokines [62]. IL-10 can also inhibits CD4^+^ T cell proliferation [62] and suppresses activation of immune effector cells such as T cells to assist cancer cell escape from immune surveillance [44]. A modest elevation of IL-10 secretion was seen in this study from macrophage interaction with C1GalT1-expressing tumour cells than with C1GalT1 suppressed cells (Fig. 6c, d). This suggests change of C1GalT1 expression by tumour cells also influences M2-mediated immune reactions in TME. Higher expression of C1GalT1 by tumour cells would therefore lead to less anti-tumour M1 type macrophages to be attracted to the tumour microenvironment for M1- macrophage-mediated anti-tumour immune reactions. This notion is supported by the discovery that more macrophages were seen in the tumour microenvironment of the ME *C1galt1*^-/-^ /Erb mice than in the *C1galt1*^f/f^/Erb mice (Fig. 7d). The demonstration of significantly lower viability of C1GalT1-suppressed tumour cells in comparison with the control cells after interaction with M1 macrophages (Fig. 6e, f) also supports this notion. Together, these results suggest a critical role of higher C1GalT1 expression by tumour cells on tumour cell avoidance of immune surveillance mediated by macrophages.

It should be mentioned that THP-1 monocytes are from the peripheral blood of a leukaemia patient. Although they have been extensively used to study monocyte/macrophage functions and are a common model to study modulation of monocyte and macrophage activities, they may not be exactly the same as the macrophages from healthy people. Further research using macrophages from healthy people will help to provide more insight into C1GalT1-medaited regulation of cancer-macrophage interactions in the tumour microenvironment.

MGL is known to be expressed by macrophages as well as DCs [42]. Like macrophages, DCs are also important components of the TME and are critically involved in immune surveillance [63]. DCs present tumour-associated antigen to T cells to aid tumour cell destruction [64]. Binding of MGL on macrophages and DC to CD45 on activated effector CD4^+^ T cells has been shown to induce reduction of effector CD4 + T cell proliferation and cell apoptosis [30, 65]. On the other hand, MGL-mediated engagement of DCs with GalNAc-containing ligands can enhance DC cell activation and survival by activation of MAPK/ERK and NF-kappaB signalling, resulting in an increase of CD8^+^ T cell mediating anti-tumour response [66] and secretion of IL-10 and TNF-α by DCs [67]. It is very possible that the altered tumour cell interaction with macrophages mediated by MGL as a result of C1GalT1 suppression shown in this study also occurs to MGL-mediated DC-tumour cell interaction and in DC-mediated immune reactions. The presence of a large number of DCs and macrophages in the tumour surroundings of ME *C1galt1*^-/-^/Erb mice compared with *C1galt1*^f/f^/Erb mice (Fig. 7c, d) is in support of this high possibility. As both macrophages and DCs play a crucial role in immune surveillance, high C1GalT1 expression in tumour cells, which leads to lower abundance of cell surface Tn antigen, would reduce MGL-mediated tumour cell interaction with macrophages and DCs. This would favour tumour cell escape of immune surveillance associated with macrophages and DCs.

Several cell membrane ion transport proteins, such as multidrug resistance-associated protein 1 (MRAP-1) and anion exchange protein-2 (AE-2), which were not known previously to be recognized by galectin-3, were extracted by galectin-3 from control but not from C1GalT1 suppressed cells. MRAP-1 is an ATP-binding cassette transporter protein that confers multiple drug resistance (e.g. to doxorubicin, vincristine and etoposide) in cancer [68]. Binding of ATP to the MRAP-1 nucleotide-binding domain causes conformational change of MRAP-1, which allows drug substrate binding on channel protein MRAP-1 to be released [68]. AE-2 is a sodium/chloride/bicarbonate transporter protein involved in the regulation of intracellular pH [69]. High AE-2 expression in ovarian cancer was shown to increase cell proliferation and was associated with low survival rate of cancer patients [70]. The extraction of these ion transport proteins by galectin-3 indicates that they carry O-linked glycans and their interaction with galectin-3 may influence ion/molecular transportation cross the cell membrane during cancer development.

A number of cell membrane proteins such as N-myristoyltransferase 1 (NMT 1), CD71, Ep-CAM and MIF are seen to be extracted by MGL differentially from C1GalT1-expressing and knockdown cancer cells in this study. CD71 is a type-II transmembrane glycoprotein that is involved in the regulation of iron uptake [71]. Ep-CAM is a key adhesion molecule of epithelial cells [72] in epithelial cell-cell interaction. The alteration of binding of these cell membrane proteins by MGL in response to change of C1GalT1 in tumour cells suggests possible involvement of these cell surface glycoproteins in MGL-mediated immune modulation in cancer.

Thus, C1GalT1 expression in cancer cells reciprocally controls tumour cell-cell and tumour-macrophage interactions mediated by galectin-3 and MGL with double impact on cancer development and progression. Higher C1GalT1 expression seen in many epithelial cancers therefore may represent a fundamental mechanism in cancer promotion and also in reduction of immune response/surveillance in cancer development and progression.

## Supplementary information

Supplementary Tables S1, S2 and Figurs S1, S2

Original Data File

check list

## Data Availability

All data generated or analysed during this study are included in this published article [and its supplementary information files].
