# Supplementary material for: C1GalT1 expression reciprocally controls tumour cell-cell and tumour-macrophage interactions mediated by galectin-3 and MGL with double impact on cancer development and progression
Source: Cell Death Dis. 2023 Aug 23;14(8):547. doi: 10.1038/s41419-023-06082-7 (PMC10447578; doi:10.1038/s41419-023-06082-7)
Supplement: Supplementary file 1 — Supplementary Tables S1, S2 and Figurs S1, S2 [file 41419_2023_6082_MOESM1_ESM.pdf]

**Table S1. Eggs with and without detectable tumour at day 14 embryonation in Chick embryo after tumour cell implantation**

| | Eggs with tumour /Total eggs | Eggs without tumour /Total eggs | p value |
|---|---|---|---|
| SW620shCon | 24/42 | 18/42 | |
| SW620shC1GT | 8/42 | 34/42 | 0.001 |
| SW620shCon+Gal3 | 8/24 | 16/24 | |
| SW620shC1GT+Gal3 | 3/24 | 21/24 | 0.011 |

**Table S2. Cell membrane proteins extracted by galectin-3 and MGL from both SW620shcon and SW620shC1GalT1 cells**

| Cell membrane proteins extracted by galectin-3 from both SW620shCon and SW620shC1GT cells | Cell membrane proteins extracted by MGL from both SW620shCon and SW620shC1GT cells |
|---|---|
| 14-3-3 protein | Annexin A1 |
| Annexin A1 | Annexin A2 |
| Annexin A2 | Annexin A3 |
| Annexin A3 | CD98 antigen |
| CD98 antigen | Clathrin heavy chain 1 |
| Ep-CAM | Complement component 1 Q subcomponent-binding protein |
| Galectin-1 | Dnj3 |
| Galectin-3-binding protein | eIF-2gX |
| HMG-2 | Extended synaptotagmin-1 |
| HMGA1 | FAU ubiquitin-like and ribosomal protein S30 |
| LAMP-1 | G25K GTP-binding protein |
| LAMP-2 | Galectin-1 |
| LCP-1 | Galectin-3 |
| MCT 4 | Galectin-3-binding protein p105 |
| Membrane-associated progesterone receptor component 1 | Magnesium transporter protein 1 |
| Moesin | MCT 1 |
| p27RF-Rho | MCT 4 |
| p59-Fyn | Moesin |
| Prohibitin-2 | Multifunctional protein ADE2 |
| Protein S100-A10 | MYH-1c |
| Protein S100-A6 | Parkinson disease protein 7 |
| Receptor of activated protein C kinase 1 | Plastin-2 |
| SLC12A2 | Protein S100-A4 |
| Spectrin alpha chain, non-erythrocytic 1 | Receptor of activated protein C kinase 1 |
| Spectrin beta chain, non-erythrocytic 1 | ZO-1 |
| Src substrate cortactin | |
| Vimentin | |

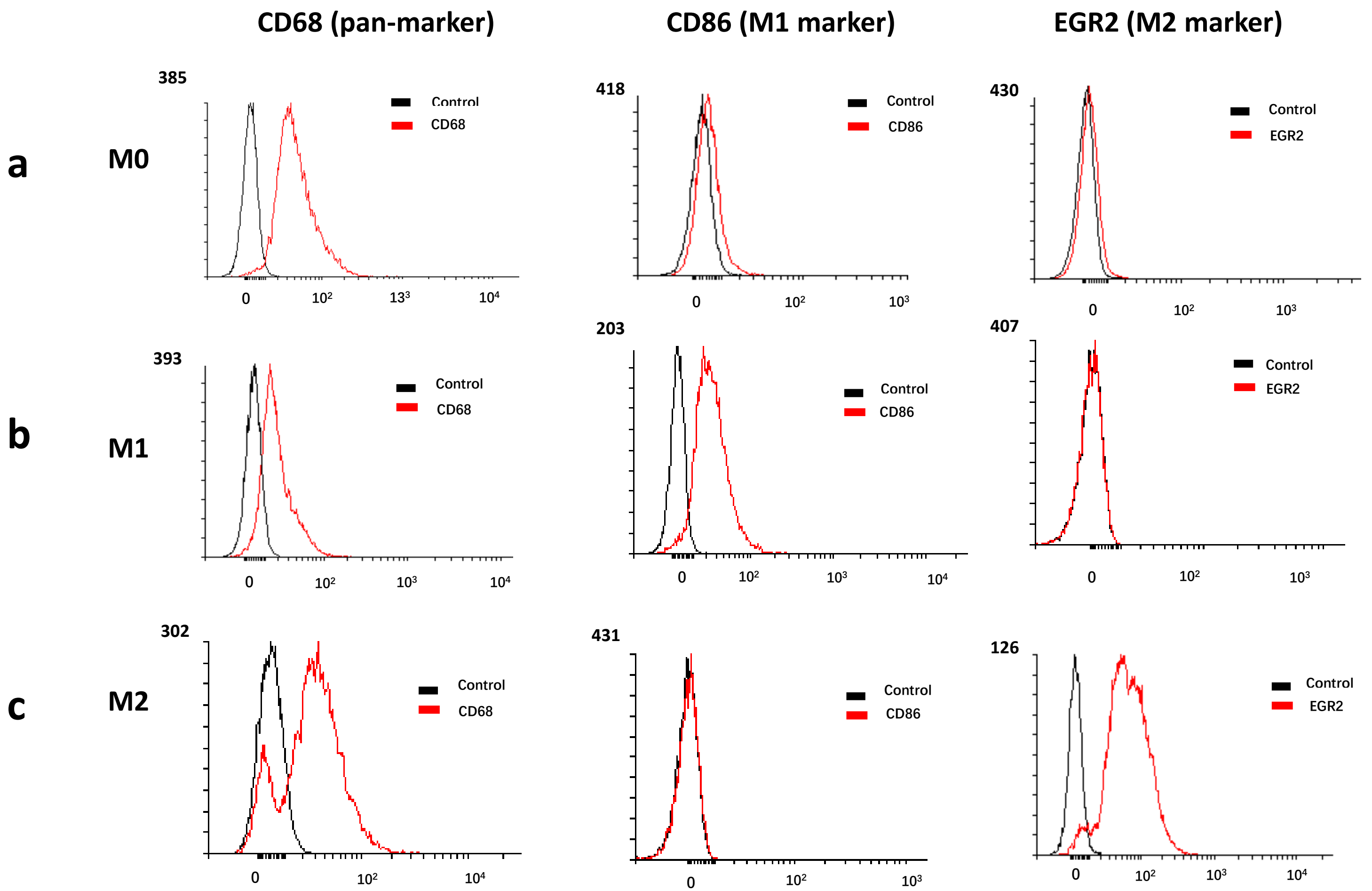


**Figure S1. Confirmation of THP-1 macrophage differentiation**. THP-1 cells were incubated with 10 ng/ml PMA for 48 h at 37°C. The cells were released with NECDS, washed once with PBS and fixed with 2% formaldehyde for 30 min. After two washes with PBS, the cells (5x$10^5$ cells/ml) were incubated with 1% BSA/PBS for 20 min before incubation with PE-conjugated antibodies against pan-macrophage marker CD68, M1 macrophage marker CD86 and M2 macrophage marker EGR2 and analysed by flow cytometry (a). THP-1 differentiated M0 macrophages were treated with 50 ng/ml IFN-γ and 100 pg/ml LPS for 24 h before analysed by flow cytometry for expression of macrophage markers (b). THP-1 differentiated M0 macrophages were treated with 60 ng/ml IL-4 for 72 h before analysed by flow cytometry for expression of macrophage markers (c). Representative flow cytometry histograms from three independent analysis are shown.

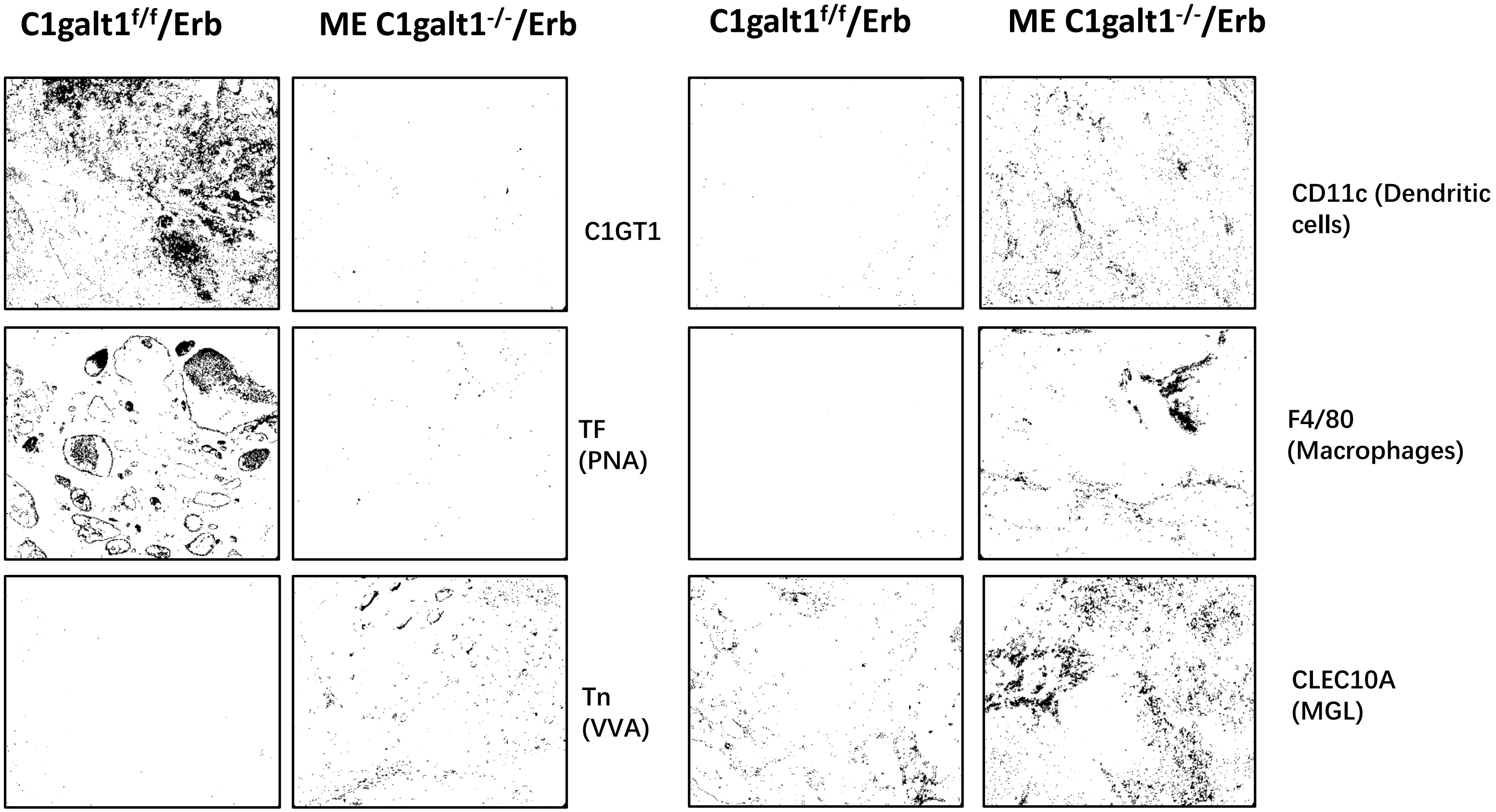


**Figure S2. Binary images in evaluation of macrophage and DC tumour infiltration.** Immunohistochemistry images of the mice sections were quantified using Fiji Image J. The final binary images of Fig 7 c (left panel) and d (right panel) were shown as example here.
