# Supplementary figures and images for: C1GalT1 expression reciprocally controls tumour cell-cell and tumour-macrophage interactions mediated by galectin-3 and MGL with double impact on cancer development and progression
Source: Cell Death Dis. 2023 Aug 23;14(8):547. doi: 10.1038/s41419-023-06082-7 (PMC10447578; doi:10.1038/s41419-023-06082-7)

Fig 1

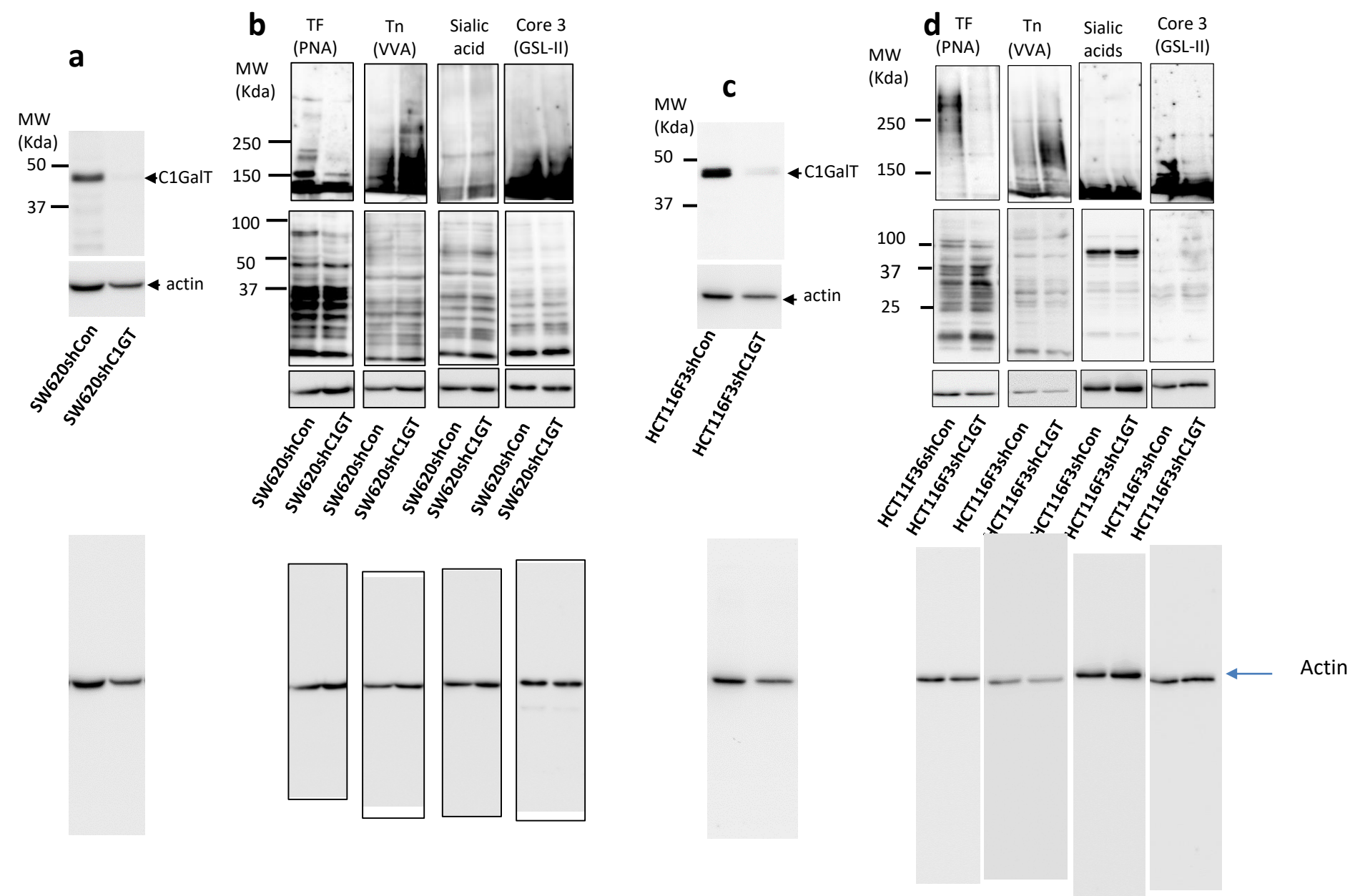

Fig 4

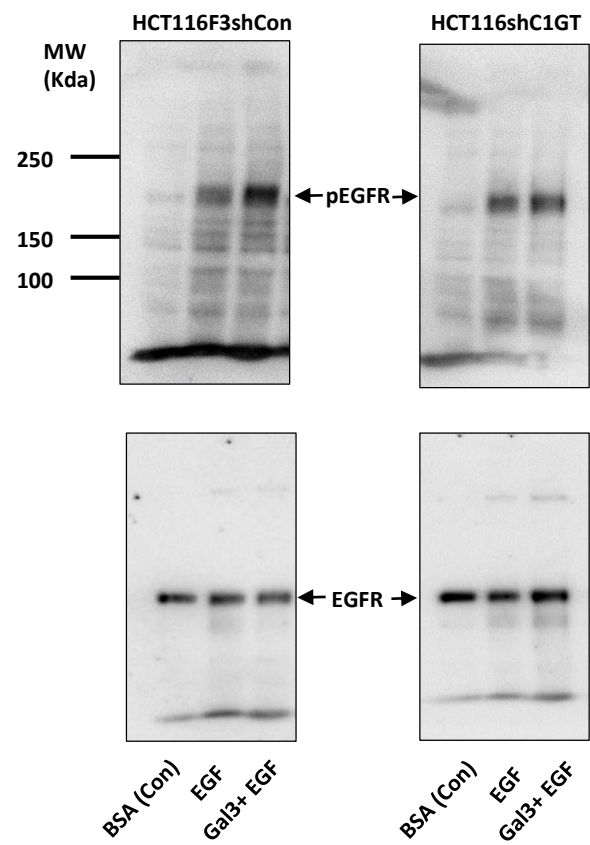

Supplement: Supplementary file 2 — Original Data File [file 41419_2023_6082_MOESM2_ESM.pdf]
